# Cuproptosis-related gene *ATOX1* promotes MAPK signaling and diffuse large B-cell lymphoma proliferation via modulating copper transport

**DOI:** 10.17305/bb.2024.10536

**Published:** 2024-07-16

**Authors:** Junjie Xie, Zhixiong Shao, Changjie Li, Cheng Zeng, Biao Xu

**Affiliations:** 1 Department of Oncology, General Hospital of Central Theater Command, Wuhan, China; 2 Jiefang Kairui Medical Laboratory, Shanghai, China; 3 Department of Hematology, General Hospital of Central Theater Command, Wuhan, China

**Keywords:** Diffuse large B-cell lymphoma (DLBCL), cuproptosis, antioxidant 1 (*ATOX1*), proliferation, copper transport

## Abstract

Diffuse large B-cell lymphoma (DLBCL) is a common subtype of non-Hodgkin lymphoma (NHL), highlighting the importance of studying susceptibility genes to develop personalized treatment strategies. While cuproptosis, caused by high levels of copper ions induced by ionophores, has been shown to affect cancer survival, its specific role in lymphoma is not yet clear. To investigate the involvement of upregulation-related genes in DLBCL, we employed bioinformatics techniques. Specifically, we analyzed the differentially expressed genes (DEGs) in the GSE25638 dataset using weighted gene co-expression network analysis (WGCNA) and performed functional enrichment analysis. By building a protein–protein interaction (PPI) network, candidate genes were identified. Gene set enrichment analysis (GSEA) and receiver operating characteristic (ROC) curve analysis were used to confirm the clinical diagnostic use of these genes. The effects of antioxidant 1 (*ATOX1*) knockdown, CuCl_2_, and DCAC50 knockdown on DLBCL cells and the activation of the mitogen-activated protein kinase (MAPK) pathway were investigated by conducting in vitro experiments. Bioinformatics and in vitro experiments confirmed elevated expression of *ATOX1* in DLBCL cells and tumor samples. *ATOX1* knockdown led to decreased cell proliferation and G2 cell cycle arrest in vitro. Additionally, phosphorylated extracellular signal-regulated kinases 1 and 2 (P-ERK1/2) protein levels within the MAPK pathway were reduced as a result of *ATOX1* knockdown, but these levels were recovered by CuCl_2_. Treatment with DCAC50 showed a dose-dependent antiproliferative effect in DLBCL cells, which was strengthened by *ATOX1* knockdown. Our study demonstrated that *ATOX1* may be important in DLBCL via controlling the MAPK pathway through copper transport, providing new insights into potential therapeutic strategies for DLBCL.

## Introduction

Lymphomas are a class of cancerous tumors that develop from lymph nodes or other lymphoid tissues [1]. These tumors are classified into two main types: non-Hodgkin lymphoma (NHL) and Hodgkin lymphoma (HL) [2, 3]. Diffuse large B-cell lymphoma (DLBCL), which makes up around 40% of all B-cell lymphomas, is the most common kind of NHL among them [4]. The most typical clinical pathological features of lymphoma are painless lymphadenopathy, hepatosplenomegaly, cachexia, fever, anemia, and swollen lymph nodes, which can become painful soon after drinking alcohol [5, 6]. Studies have shown that immunocompromise, genetics, smoking, and infectious factors, such as Epstein–Barr (EB) virus infection, retrovirus, human herpes virus, measles virus, and *Helicobacter pylori* infection are all risks of lymphoma [7, 8]. Due to the high heterogeneity of lymphoma, the treatment effect varies widely, and the prognosis of patients is often related to different pathological types and stages. Therefore, exploring the susceptibility genes of lymphoma based on molecular biology is of much significance for clinical diagnosis and the development of individualized treatment plans.

Copper can induce a new mode of cell death called copper-dependent apoptosis (cuproptosis), which is associated with excessive copper ion concentrations [9]. Copper is an essential micronutrient that is required for various physiological processes in almost all cell types. However, excessive accumulation of intracellular copper induces oxidative stress and disrupts cellular function, so copper homeostasis is tightly regulated. Recent studies have identified a new form of copper-dependent cell death, which is distinct from all other known cell death pathways. Copper-dependent apoptosis occurs through the binding of copper to the enzyme thioctanoylase in the tricarboxylic acid (TCA) cycle, which leads to subsequent protein aggregation, proteotoxic stress, and ultimately cell death [10]. During this process, copper ionophore is a small molecule that can bind with copper and transport it into cells, and some copper ionophores are even used for cancer treatment [11, 12]. It has been demonstrated that cuprotosis affects the survival prognosis in a number of malignancies. One of the important regulators, *FDX1*, has been demonstrated to be substantially downregulated in hepatocellular carcinoma (HCC), higher expression of this gene is associated with a longer survival period [13]. Cuproptosis is a newly identified form of copper-driven cell death that has attracted much attention in recent years in the study of cancer pathogenesis. Due to the important role of copper and its triggered cell death in tumorigenesis, copper-based therapies show the potential to inhibit tumor growth, especially in response to tumors that are insensitive to chemotherapy and may offer new strategies for cancer treatment [14]. However, it is still necessary to investigate the mechanism by which cuprotosis genes cause lymphoma.

The mitogen-activated protein kinase (MAPK) cascade is a key signaling pathway that regulates a variety of cellular processes, including proliferation, differentiation, apoptosis, and stress responses. The MAPK pathway consists of three major kinases: the MAPK kinase kinase, the MAPK kinase, and the MAPK, which activate and phosphorylate downstream proteins sequentially [15]. Activation of the mitogenic signaling pathway is a common oncogenic driver of many solid tumors, recent study has shown that selective inhibition of the MAPK pathway can suppress ASCL1-driven small cell lung cancer [16]. In addition, recent sequencing and transcriptomics studies have demonstrated the important contribution of the RAS-MAPK pathway to the development and progression of neuroblastoma [17]. The MAPK pathway plays a key role in oxidative stress, while AMPK acts as a sensor of cellular energy and participates in the regulation of energy stress response. Activation of AMPK not only induces autophagy-dependent iron death but also activates iron death via p53, which provides a new direction for the future study of the mechanism of iron death and brings a new vision for cancer treatment strategies [18]. In addition, it was found that cadmium induced apoptosis and mitochondrial damage in human bronchial epithelial cells (BEAS-2B) through the MAPK signaling pathway [19]. These studies showed that the MAPK pathway has an important regulatory role in a variety of cellular activities and disease processes, providing new potential targets for the treatment of cancer and other diseases.

In this study, we employed bioinformatics approaches to analyze the GSE25638 dataset using 129 cuproptosis-related genes obtained from the MSigDB, which is part of the gene set enrichment analysis (GSEA) platform, identifying *ATOX1* as the pivotal cuproptosis-related gene. Subsequently, we explored the regulatory interactions between *ATOX1* and the MAPK pathway in DLBCL cells using in vitro experiments. We also assessed the impact of CuCl_2_ and DCAC50 treatments on DLBCL cell growth and the MAPK signaling pathway. Through these investigations, we aim to offer novel insights into therapeutic strategies for DLBCL management.

## Materials and methods

### Acquisition and analysis of the GSE25638 dataset

The Gene Expression Omnibus (GEO) database (https://www.ncbi.nlm.nih.gov/geo/) provided the DLBCL-related GSE25638 dataset, which included 97 samples in total. Of these, 13 normal B cell purified subpopulation samples and 26 DLBCL samples were used for the investigation. The selection of the 13 normal B cell subpopulation samples was made to represent the diversity of normal B cells, while the choice of 26 DLBCL samples aimed to cover the different subtypes or characteristics of DLBCL. It should be noted that this investigation did not include validation of the findings using an independent cohort. Differentially expressed genes (DEGs) that were upregulated and downregulated were screened using the GEO2R program (specifically, 13 normal B cell purified subpopulation samples and 26 DLBCL samples). The criteria were log_2_ fold change (log_2_FC)>1 for upregulation and <−1 for downregulation, both with *P* < 0.05.

### Weighted gene co-expression network analysis (WGCNA)

To find the optimal soft threshold, a gene co-expression network based on the DEGs screened in the GSE25638 dataset was constructed using the WGCNA method (signed hybrid). The genes were then separated into modules of various colors. The key module was determined by comparing the correlation between the modules and the two sets of samples in the GSE25638 dataset, generating the cluster dendrogram between the modules and the feature gene adjacency heatmap between the modules. In order to further determine the key modules related to DLBCL, the relationship between module eigengenes (MEs) and samples was calculated by the Pearson correlation coefficient.

### Enrichment analysis of genes in the turquoise module

To visualize the gene expression patterns of the turquoise module, a heatmap was generated depicting the expression levels of genes within this module. The Database for Annotation, Visualization, and Integrated Discovery (DAVID; https://david.ncifcrf.gov/tools.jsp) was used to analyze key modules. The Kyoto Encyclopedia of Genes and Genomes (KEGG) pathway database and the Gene Ontology (GO) categorization, which includes biological processes (BPs), cellular components (CCs), and molecular functions (MFs), were both employed in the enrichment study. When *P* < 0.05, the obtained enrichment results were considered statistically significant.

### Protein–protein interaction (PPI) networks of cuproptosis-related genes

By utilizing GSEA (https://www.gsea-msigdb.org), we identified these genes associated with cuproptosis. Subsequently, using the R software package “VennDiagram,” we identified genes common to the turquoise module, which exhibited the strongest correlation with the samples, and a set of 129 cuproptosis-related genes. These overlapping genes were then uploaded to the STRING database (https://string-db.org/) to obtain interaction information. The resulting data were visualized using Cytoscape software to construct and visualize the PPI network. To prioritize genes within this network, the Cytohubba plug-in in Cytoscape was employed, applying the MCC, BottleNeck, and Degree algorithms separately to the overlapping genes. Detailed steps involved accessing the STRING database online to download files compatible with Cytoscape for further analysis (separately access the STRING database online and download files to open them using Cytoscape). Only the top ten genes in each subnetwork were then chosen, and candidate genes were once more found using the “VennDiagram” package of R software.

### Functional enrichment and diagnostic potential of candidate genes in DLBCL

To delineate the functional significance of the six candidate genes, we performed a GSEA single-gene Wikipathway enrichment analysis, selecting outcomes with a significance threshold set at *P* < 0.05. After that, receiver operating characteristic (ROC) curves were drawn for these genes using the R software’s “timeROC” function. The area under the curve (AUC) values obtained from the ROC analyses were used to critically evaluate the diagnostic predictive accuracy of these genes. Moreover, the Gene Expression Profiling Interactive Analysis2 (GEPIA2; http://gepia2.cancer-pku.cn) database was used to examine the expression profile of *ATOX1* (key gene) in DLBCL.

### Cell culture, treatment, and transfection

From the American Type Culture Collection (ATCC), we obtained three DLBCL cell lines (OCI-LY7, DB, and U2932) as well as the B-lymphocyte cell line (HCC38BL). The RPMI 1640 medium with 10% fetal bovine serum (FBS, Gibco) and 1% penicillin–streptomycin solution (Solarbio, China) was used to cultivate these cells. The cells were maintained at 37 ^∘^C and 95% relative humidity in a humidified environment with 5% CO_2_. 10 µM CuCl_2_ and various concentrations of DCAC50 (a newly discovered small molecule inhibitor of the intracellular copper chaperones *ATOX1*) (1, 2.5, 5, 10, 15, and 20 µM) were applied to the DB and U2932 cell lines (high *ATOX1* expression), respectively. Two different small interfering RNAs (siRNAs), denoted as si-*ATOX1* #1 and si-*ATOX1* #2, were used to knock down *ATOX1* expression in gene silencing assays. Transfections were carried out using the Lipo2000 reagent (Polyplus Transfection, France), following the manufacturer’s instructions. The control group consisted of cells transfected with non-targeting control siRNA, often referred to as si-negative control (NC).

### Quantitative reverse transcription polymerase chain reaction (qRT-PCR) assay

With the use of the cDNA synthesis kit (DBI, Germany) and Trizol reagent (Invitrogen, USA), we were able to extract the RNA from transfected cells and perform reverse transcription. SYBR Green Master Mix (Applied Biosystems, USA) was used for qRT-PCR, which was carried out using an AB Fast 7500 real-time apparatus. For amplification, the primer sequences listed in the Table S1 were used. After standardizing the expression levels against the internal reference GAPDH [20], the relative gene expression levels of *ATOX1* were calculated using the 2^−ΔΔCt^ approach.

### Western blotting (WB) assay

Protease and phosphatase inhibitors (Roche) were added to RIPA lysis buffer (Beyotime Biotechnology) to aid in the extraction of proteins from transfected cells. The BCA protein assay kit (Thermo Fisher Scientific) was used to measure the concentrations of proteins. Using SDS-PAGE, proteins were divided into equal portions and then transferred to Millipore PVDF membranes. Next, primary antibodies (1:1000, Abcam, USA) and GAPDH (1:5000, Abcam, USA) were probed overnight at 4 ^∘^C on the membrane. The primary antibodies included *ATOX1*, p27, Cyclin B1, P-MEK1/2, T-ERK1/2, T-MEK1/2, P-MEK1/2, T-p38, P-p38, T-JNK, and P-JNK. After washing, the membrane was incubated for an hour at room temperature with an HRP-conjugated secondary antibody (1:5000, Abcam, USA). Enhanced chemiluminescence (ECL) substrates from Bio-Rad were visualized to identify protein bands, the ChemiDoc™ Imaging System from the same manufacturer was utilized to detect the bands.

### Flow cytometry

Flow cytometry was used to evaluate the cell cycle distribution in DLBCL cells. Propidium iodide (PI) staining was applied to cells by a standard protocol, the cells were then analyzed using a BD FACSCanto II flow cytometer. Data interpretation and cell cycle phase quantification were executed using appropriate analytical software.

### Cell Counting Kit-8 (CCK-8)

The CCK-8 kit (Qihai Biotechnology, China) was used to test cell proliferation. 96-well plates were used to cultivate the transfected and DCAC50-treated cells after incubating them in CCK-8 solution for two to four hours at 37 ^∘^C. The absorbance (OD) of each well was measured at 450 nm after 24, 48, and 72 h of incubation.

### Statistical analysis

The data was analyzed using GraphPad Prism (version 9.0.0, GraphPad Software, USA). All experiments were repeated at least three times and data are presented as mean ± standard error. To establish statistical significance, the student’s *t*-test or one-way analysis of variance (ANOVA) were performed, then the post hoc Tukey multiple comparison test was used to assess the results. The statistically significant result was defined as *P* < 0.05.

**Figure 1.**
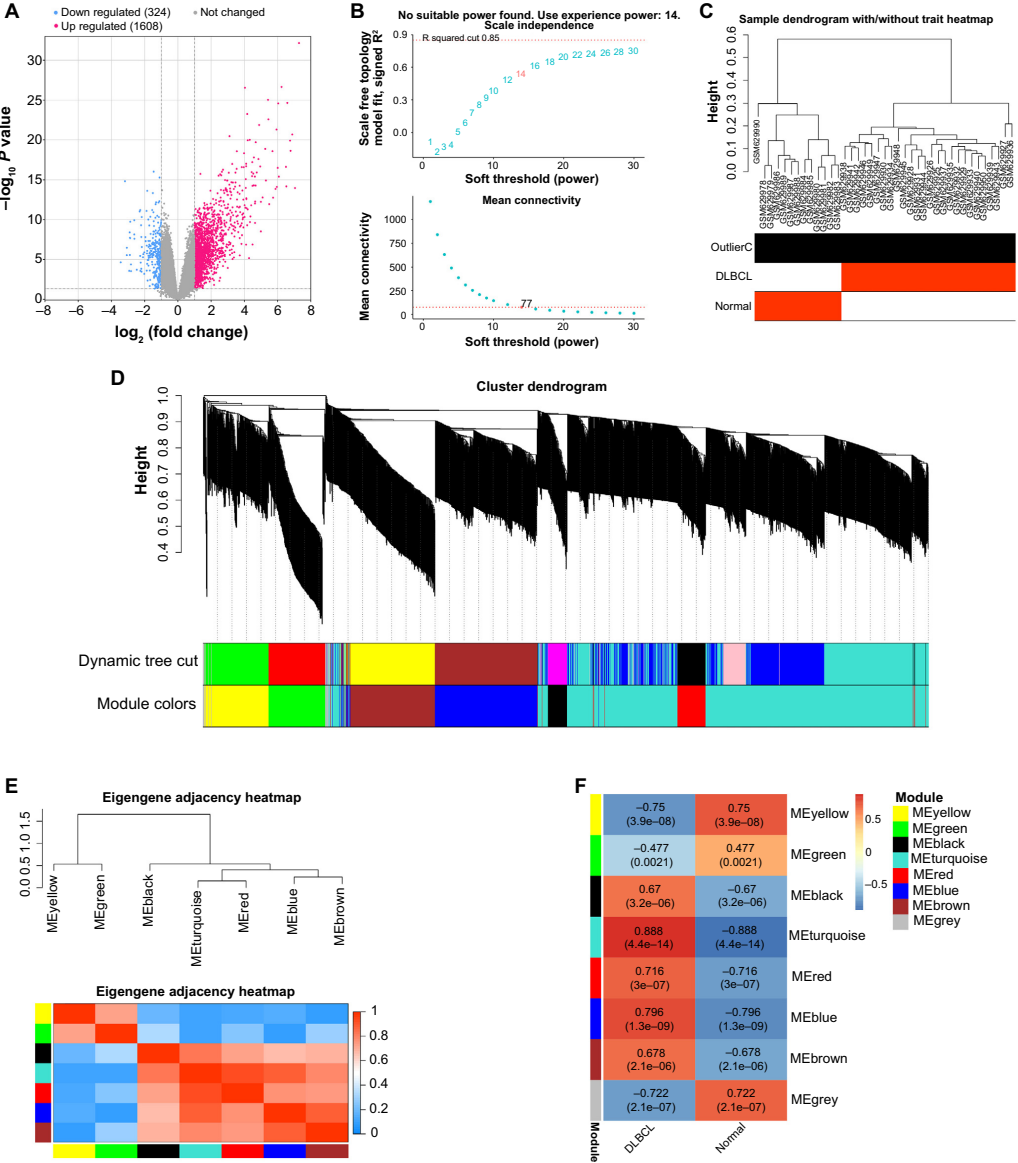
**Gene co-expression network of DEGs in the GSE25638 dataset.** (A) Volcano plot, red scatter points represent upregulated DEGs in the GSE25638 dataset, and blue scatter points represent downregulated DEGs in the GSE25638 dataset; (B) The picture above is the reference picture for the best soft threshold, the red line represents the subjectively selected scale-free fitting index, the picture below shows the mean connectivity corresponding to different soft thresholds; (C) Sample dendrogram of the GSE25638 dataset, with different branches representing different samples; (D) The upper part is the hierarchical clustering dendrogram of genes, the lower part is the gene modules with different colors; (E) Eigengene adjacency heatmap between gene modules; (F) Heatmap of the correlation between gene modules and clinical samples, the color on the left represents the module, the middle is the correlation between the module and the sample, the color on the right represents the range of correlation. DEGs: Differentially expressed genes; DLBCL: Diffuse large B-cell lymphoma.

## Results

### Screening of DEGs in DLBCL and identification of the turquoise module

We utilized the GEO2R tool from the GSE25638 dataset to detect 1608 upregulated and 324 downregulated DEGs (Figure 1A). By applying the WGCNA algorithm with a soft threshold of 14, we constructed a gene co-expression network for these DEGs (Figure 1B). Next, we classified the genes into distinct color modules based on their expression levels, depicted in Figure 1C and 1D. Evaluating the correlation between the DLBCL and normal samples, we observed a significant relationship (correlation coefficient of 0.888) between the turquoise module and the samples (*P* value 4.4e-14) (Figure 1E and 1F). As a result, we identified the turquoise module as the pivotal module.

### Functional enrichment analysis reveals key BPs and pathways of turquoise module

**Figure 2.**
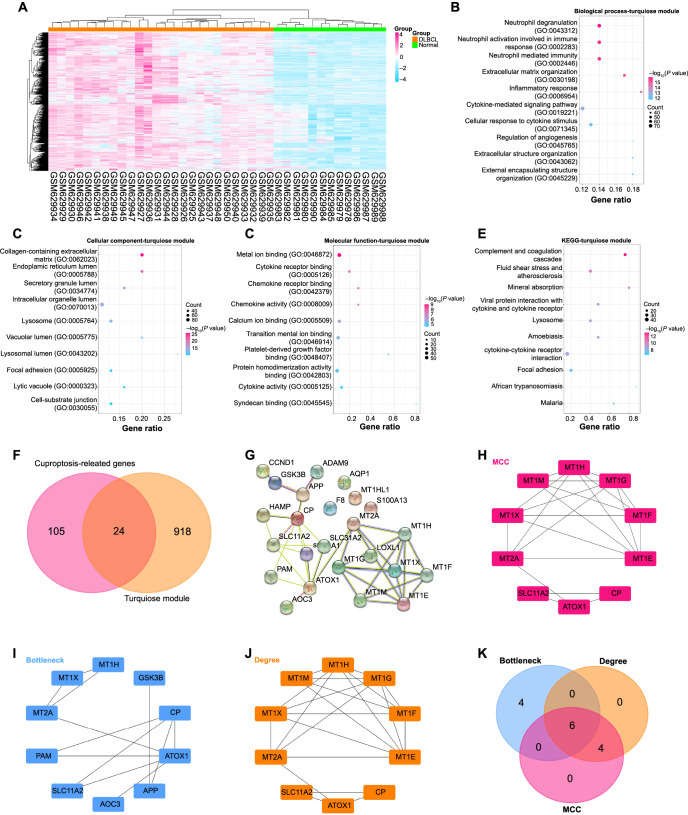
**Turquoise module and cuproptosis-related genes revealed six candidates through the PPI network.** (A) Heatmap of the cluster distribution of genes in the turquoise module in the GSE25638 dataset, with 26 DLBCL samples listed in red and 13 normal control samples listed in green; (B–E) Bubble plots showing the top ten enriched terms for different categories: BP, CC, MF, and KEGG pathway. Each bubble represents a term and its size is proportional to the number of enriched DEGs in that term. Colors indicate the significance level of enrichment, with darker shades representing higher significance; (F) Venn diagram, the middle part is the 24 genes that intersect cuproptosis-related genes and DEGs from GSE25638; (G) The PPI network of 24 genes constructed from the STRING database with 24 nodes and 37 edges; (H–J) Sub-networks of top ten genes constructed by the MCC, BottleNeck and Degree algorithms in the Cytohubba plugin; (K) Venn diagram screened out six intersection genes in MCC, BottleNeck, and Degree algorithm. DLBCL: Diffuse large B-cell lymphoma; DEGs: Differentially expressed genes; PPI: Protein–protein interaction; BP: Biological process; CC: Cellular component; MF: Molecular function; *ATOX1*: Antioxidant 1.

The turquoise module had a total of 942 genes (Figure 2A), which were subjected to GO and KEGG pathway enrichment analysis. As shown in Figure 2B–2D, the enriched terms of these DEGs in BP, CC, and MF included, e.g., Neutrophil degranulation, Neutrophil-mediated immunity, Endoplasmic reticulum lumen, and Metal ion binding. Furthermore, KEGG pathways, such as Malaria, cytokine–cytokine receptor interaction, focal adhesion, complement and coagulation cascades, and lysosome were enriched in these DEGs (Figure 2E).

### Turquoise module and cuproptosis-related genes revealed six candidates through the PPI network

We identified 24 overlapping genes from a pool of 942 GSE25638-DEGs and 129 cuproptosis-related genes. These genes formed a PPI network consisting of 24 nodes and 37 edges (Figure 2F and 2G). The Cytohubba plug-in in the Cytoscape program was then utilized to generate three sub-networks using the MCC, BottleNeck, and Degree algorithms (Figure 2H–2J). By comparing the results from these three topological analysis methods using Venn diagrams, six central candidate genes were identified (Figure 2K).

### *ATOX1* was the hub gene related to cuproptosis in DLBCL

**Figure 3.**
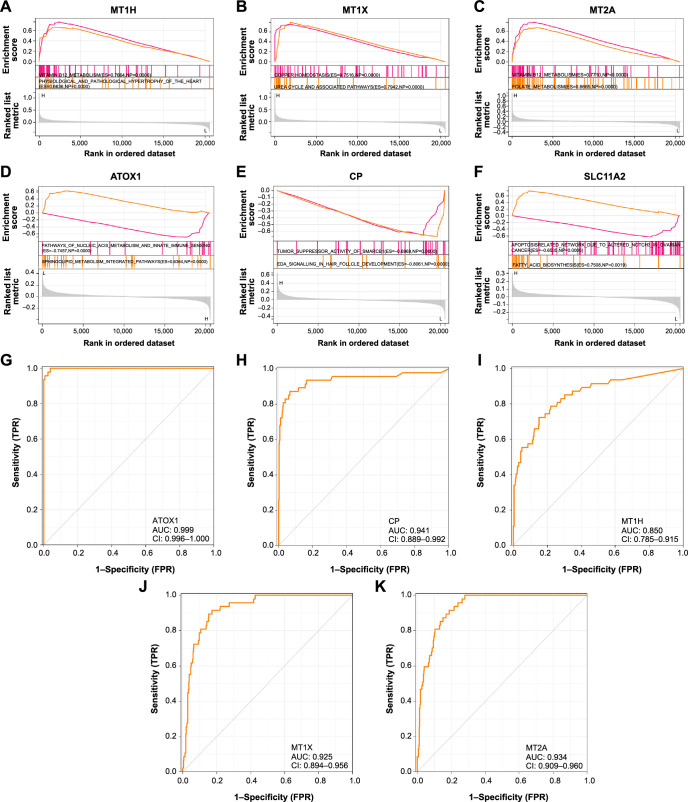
**The GSEA and ROC analysis on the candidate genes.** (A–F) Enrichment analysis on six candidate genes using WikiPathway module from GSEA. The *x*-axis represents rank in an ordinal dataset, while the *y*-axis shows ranked list metrics. Enrichment scores are represented by colored bars, indicating the importance of the enriched term and its association with the dataset; (G–K) The ROC curves of the five significant candidate genes. The AUC value represents the ability of the model to distinguish positive from negative cases, where higher AUC values indicate better predictive accuracy. GSEA: Gene set enrichment analysis; ROC: Receiver operating characteristic; AUC: Area under the curve; ATOX1: Antioxidant 1.

**Figure 4.**
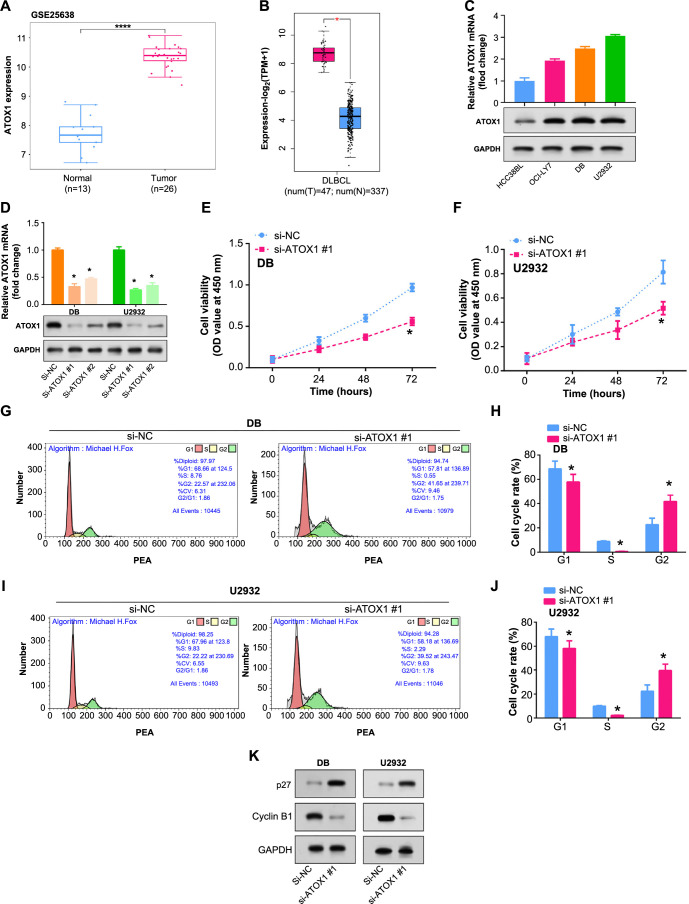
**Regulatory effects of *ATOX1* knockdown on cell proliferation, and cell cycle progression in DLBCL cells.** (A) Expression levels of copper apoptosis-related gene *ATOX1* in DLBCL samples and normal samples in the GEPIA2 database; (B) Expression levels of *ATOX1* in DLBCL cells and control HCC38BL determined by qRT-PCR (upper parts) and WB (bottom parts) assays; (C and D) qRT-PCR (upper parts) and WB (bottom parts) assays were used to determine the expression level of *ATOX1* in DLBCL cells after knocking down *ATOX1*; (E and F) CCK-8 detected the regulatory effect of *ATOX1* knockdown on DLBCL cell proliferation; (G–J) Flow cytometry analysis depicting the cell cycle distribution of DLBCL cells after knockdown of *ATOX1* #1. Histograms show the distribution of cells in different phases of the cell cycle including G1 (Gap 1), S (Synthetic), and G2 (Gap 2); (K) WB detection of knockdown *ATOX1* #1 cell cycle-related protein expression in DB and CYP6. *P < 0.05. DLBCL: Diffuse large B-cell lymphoma; GEPIA2: Gene Expression Profiling Interactive Analysis2; qRT-PCR: Quantitative reverse transcription polymerase chain reaction; WB: Western blotting; CCK-8: Cell counting kit-8; *ATOX1*: Antioxidant 1.

GSEA was performed using Wikipathway module/category on six genes, each gene was enriched with two pathways (Figure 3A–3F). GSEA showed MT1H enrichment in Vitamin B12 metabolism and physiological and pathological hypertrophy of the heart pathways (Figure 3A), MT1X was enriched in Copper homeostasis and Urea cycle and association pathways (Figure 3B), MT2A showed enrichment in Vitamin B12 metabolism and Folate metabolism pathways (Figure 3C), *ATOX1* was enriched in Pathways of nucleic acid metabolism and Sphingolipid integrated pathway (Figure 3D), CP showed enrichment in Tumor suppressor activity of smarcb1 and EDA signaling in hair follicle development pathways (Figure 3E), SLC11A2 was enriched in apoptosis-related network due to altered notch3 in ovarian cancer and Fatty acid biosynthesis pathways (Figure 3F). Then in ROC curve analysis (all patients), *ATOX1* had the highest AUC value of 0.999 (Figure 3G), indicating that it had the strongest predictive ability for DLBCL patients, followed by CP (Figure 3H, AUC ═ 0.941), MT2A (Figure 3I, AUC ═ 0.934), MT1X (Figure 3J, AUC ═ 0.925), MT1H (Figure 3K, AUC ═ 0.850). These findings showed that *ATOX1*, CP, MT2A, MT1X, and MT1H could be the diagnostic biomarkers for DLBCL patients. GEPIA database showed that *ATOX1* gene was highly expressed in the tumor tissues of DLBCL (Figure 4A). In this study, we selected *ATOX1* as the hub gene for subsequent analysis.

### *ATOX1* depletion induces G2 cell cycle arrest and alters cell cycle regulatory proteins in DLBCL

We used qRT-PCR and WB assays to identify *ATOX1* expression in DLBCL cells. When compared to the control cell HCC38BL, *ATOX1* mRNA and protein levels were considerably higher in DB, and U2932 cells in DLBCL, where DB and U2932 exhibited significant upregulation (Figure 4B). Knockdown using si*-ATOX1#*1 showed greater efficiency in DB and U2932 cell lines and was selected for subsequent analysis (Figure 4C). *ATOX1*#1 knockdown dramatically lowered the proliferation rate of DLBCL cells, as demonstrated by CCK-8 assays (Figure 4D and 4E). The results from flow cytometry indicated that when *ATOX1* was knocked down using si-*ATOX1*#1, there was a notable difference compared to the control group, further investigation into the cell cycle demonstrated a significant G2 phase arrest (Figure 4F and 4G). WB methods were utilized to determine the protein expression levels of cell cycle-related proteins in DLBCL cell lines following *ATOX1* knockdown. The results showed that after knocking out *ATOX1*, cell cycle regulatory protein (Cyclin B1) was significantly downregulated, while cell cycle negative regulatory protein (p27) was significantly upregulated (Figure 4H). This supported a potential role for *ATOX1* in cell cycle control in DLBCL.

### *ATOX1* knockdown and copper supplementation impact MAPK pathway protein expression in DLBCL cells

**Figure 5.**
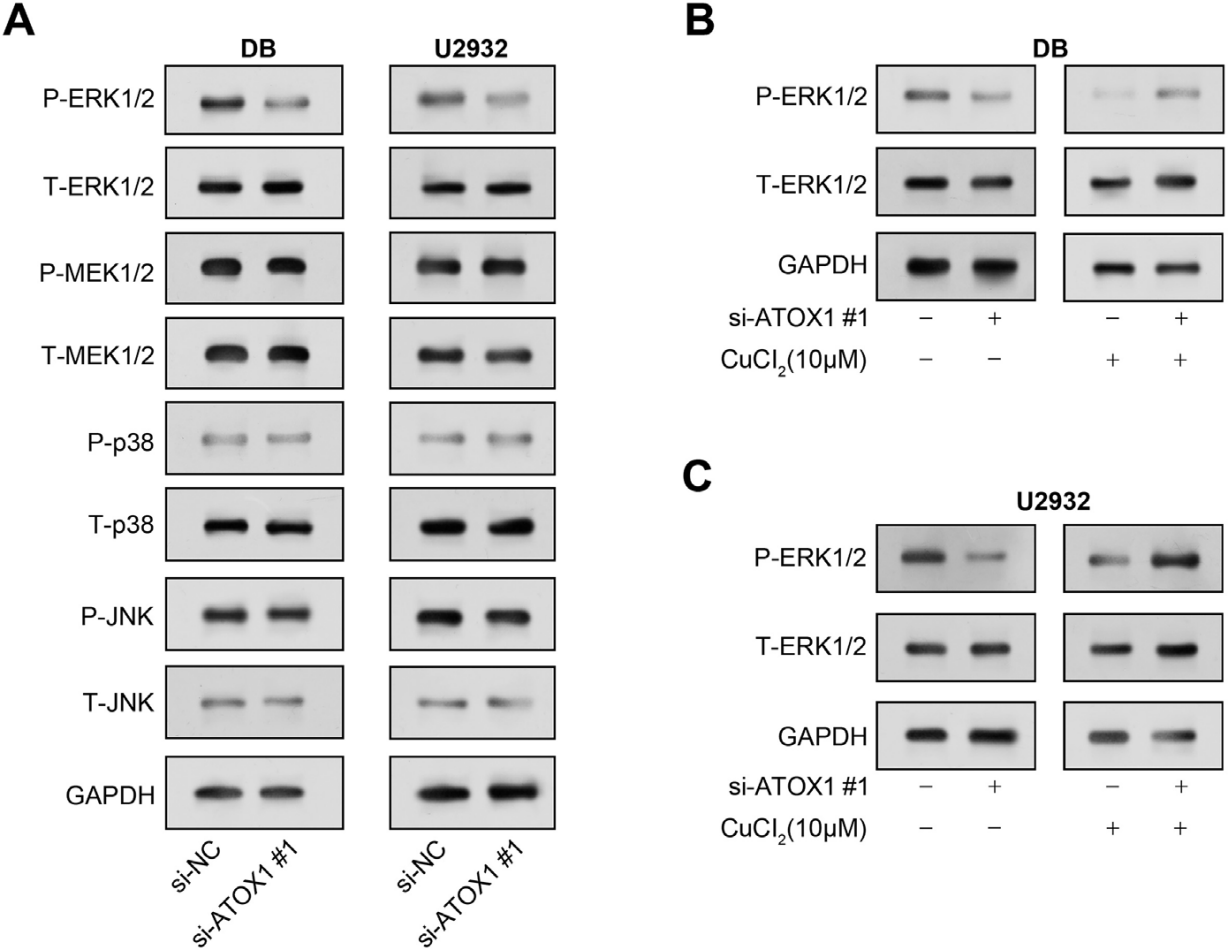
**Modulation of MAPK pathway protein levels following *ATOX1* knockdown and CuCl_2_ treatment.** (A) WB detection of MAPK pathway protein levels in DLBCL cells after *ATOX1* knockdown; (B and C) After knocking out *ATOX1* and adding 10 µM CuCl_2_, the protein levels of some members of the MAPK pathway in DLBCL cells were detected by WB. DLBCL: Diffuse large B-cell lymphoma; MAPK: Mitogen-activated protein kinase; WB: Western blotting; *ATOX1*: Antioxidant 1.

The MAPK pathway is a signaling cascade that regulates a number of cellular functions, including the progression of lymphoma. In the MAPK pathway, P-ERK1/2 is crucial for regulating a number of BPs, including cell division, proliferation, and survival. We continued to use the WB method to detect the protein level of the MAPK pathway after knocking down *ATOX1* in DLBCL cells. The findings demonstrated that following *ATOX1* knockdown, P-ERK1/2 components of the MAPK pathway had much lower levels of protein expression in DLBCL cell lines (Figure 5A). It was found that reducing *ATOX1* expression led to a decrease in the expression levels of proteins in the MAPK pathway could be reversed by knocking down *ATOX1* and adding 10 µM CuCl_2_ (Figure 5B and 5C). This showed that in DLBCL cells, the activation of the MAPK pathway may be modulated by *ATOX1* and copper.

### Inhibitory effects of DCAC50 treatment and *ATOX1* knockdown on DLBCL cell proliferation and MAPK pathway

**Figure 6.**
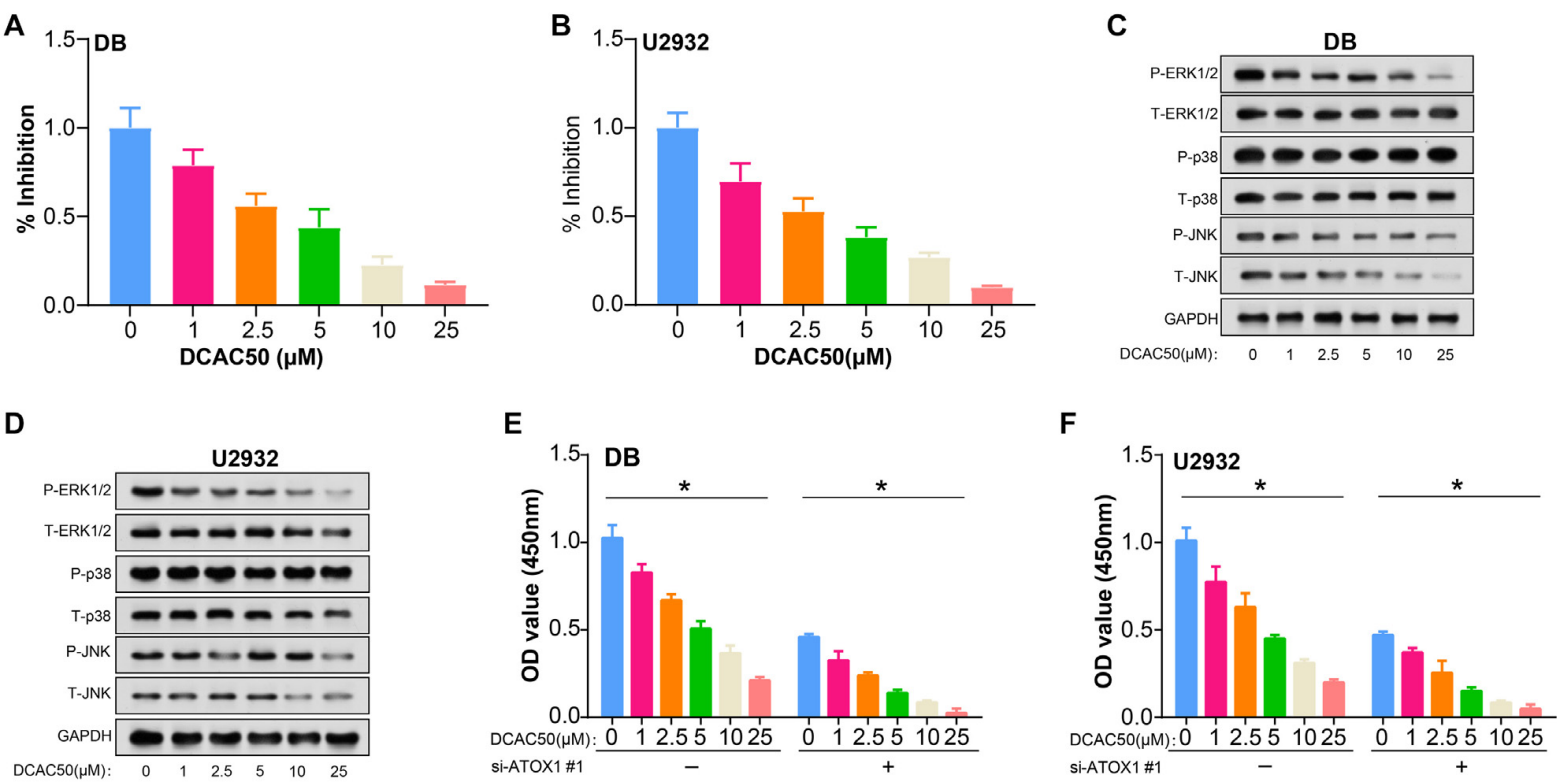
**Evaluation of DLBCL cell responses to DCAC50 treatment and *ATOX1* knockdown.** (A and B) IC50 by CCK-8 assay of DLBCL cells treated with different concentrations of DCAC50 (0, 1, 2.5, 5, 15, and 20 µM); (C and D) The impact of DCAC50 gradient dose treatment (0, 1, 2.5, 5, 15, and 20 µM) on the levels of MAPK proteins evaluated using western blot; (E and F) CCK-8 detects the effect of *ATOX1* knockdown and DCAC50 gradient dose treatment (0, 1, 2.5, 5, 15, and 20 µM) on the proliferation of DLBCL cells. *P < 0.05. DLBCL: Diffuse large B-cell lymphoma; MAPK: Mitogen-activated protein kinase; CCK-8: Cell counting kit-8; *ATOX1*: Antioxidant 1.

The newly discovered small molecule inhibitor, DCAC50, reduces cell proliferation and increases oxidative stress by targeting intracellular copper chaperones *ATOX1* and CCS. We assessed cell IC50 of DLBCL cells using a CCK-8 assay after treatment with different concentrations of DCAC50. Treatment with DCAC50 significantly inhibited cell activity compared with untreated DLBCL cells. Notably, the cells treated with 15 and 20 µM DCAC50 showed the largest decrease in cell IC50 (Figure 6A and 6B). WB analysis showed that protein levels were noticeably reduced for most members of the MAPK pathway in DLBCL cells treated with DCAC50, indicating potential disruption of this pathway and contributing to its anti-proliferative effects (Figure 6C and 6D). Moreover, the simultaneous knockdown of *ATOX1#1* enhanced the inhibitory effects of DCAC50 on cell proliferation (Figure 6E and 6F). These findings suggest that DCAC50 exerts a dose-dependent inhibitory effect on DLBCL cell growth, with more pronounced effects observed at higher concentrations, and knockdown of *ATOX1*#1 promoting its effects.

## Discussion

The most common form of malignant lymphoma in medical practice is DLBCL, which primarily affects middle-aged and elderly individuals [21]. Studies have demonstrated that typical indications of DLBCL include high fever and a range of systemic symptoms, such as an enlarged painless neck and progressive enlargement of lymph nodes in the supraclavicular region [22, 23]. Furthermore, personalized chemotherapy combined with immune-targeted therapy is often utilized based on specific symptoms, type of pathology, and location of the tumor. However, due to its high prevalence and aggressiveness, the treatment of DLBCL is often challenging and the prognosis is usually poor [24, 25]. Previous research has proposed that cuproptosis may contribute to the development of malignant tumors [26, 27]. In this study, we employed bioinformatics and in vitro experimental investigation to examine the mechanism and clinical significance of cuproptosis-related genes in DLBCL.

We conducted a gene co-expression network analysis using WGCNA on the GSE25638-DEGs obtained from the GSE25638 database. Results showed that the turquoise module was the most significant in this network. Subsequently, we performed functional analysis on the genes within the turquoise module and identified enrichment in cytokine receptor binding, amoebiasis, cytokine–cytokine receptor interaction, focal adhesion, and malaria. These pathways have been previously linked to the development of lymphoma. For instance, Waldron et al. [28] proposed that cytokine–receptor interactions play a role in the biology of Hodgkin’s disease and anaplastic large cell lymphoma, differentiating them from other forms of lymphoma. According to research by Bosch et al. [29], focal adhesion is linked to a bad prognosis in DLBCL patients, the expression of the related protein FAK, may be an independent prognostic factor for DLBCL. Furthermore, studies have shown a connection between malaria infection and endemic Burkitt lymphoma [30]. In summary, our examination underscores the importance of pivotal gene modules in DLBCL, indicating their possible part in the etiology and outcome of the illness, which is corroborated by extant research.

Based on this, we identified six candidate genes (*ATOX1*, *CP*, *MT1H*, *MT1X*, *MT2A*, and *SLC11A2*) from GSE25638-DEGs and cuproptosis-related genes by PPI networks, verified their clinical value in DLBCL. The GSEA-Wikipathway results showed that the enrichment items of these genes included Copper homeostasis, Vitamin B12 metabolism, Fatty acid biosynthesis, etc. Several studies have shown that vitamin B12 intake has a protective effect on NHL in heavy smokers [31, 32]. Fatty acids can affect cancer cell invasion and lymph node metastasis, their oxidative pathways provide new targets for the treatment of DLBCL [33]. As a treatment option for DLBCL, fatty acid synthase inhibition of fatty acid synthase preferentially disrupts de novo fatty acid synthesis [34]. According to ROC analysis, *ATOX1*, CP, MT2A, MT1X, and MT1H all had AUC values better than 0.85. The AUC value of *ATOX1* was the highest, indicating that it had the strongest predictive ability for DLBCL patients. Furthermore, GEPIA database analysis revealed that the *ATOX1* gene was substantially expressed in DLBCL tumor tissues. As a result, we chose *ATOX1* as the hub gene.

*ATOX1*, a copper chaperone, has a significant impact on cellular antioxidant defense and copper homeostasis [35]. It is involved as a cytoplasmic protein in the transport of intracellular copper ions to various copper-dependent enzymes, including those involved in oxidative stress protection and angiogenesis [36]. *ATOX1* is responsible for delivering copper to copper-dependent enzymes involved in antioxidant defense and extracellular matrix remodeling. *ATOX1*-mediated dysregulation of copper homeostasis is associated with increased oxidative stress and altered cellular behavior, potentially leading to cancer development [37]. Research has indicated that breast cancer cell migration is influenced by the copper chaperone *ATOX1* [38]. Another study showed that activin A-induced migration and colony formation of colon cancer cells were enhanced by nuclear translocation of *ATOX1* [39]. Through a series of in vitro cell experiments, we discovered that the knockdown of *ATOX1* inhibited cell growth and caused cell cycle arrest in the G1 phase of the cell cycle. Knockdown of *ATOX1* resulted in downregulation of Cyclin B1 protein expression level and upregulation of p27 protein expression, implying its role in inhibiting cell cycle progression. Moreover, we found that after *ATOX1* knockdown, P-ERK1/2 levels within the MAPK pathway significantly decreased. Notably, copper supplementation partially restored MAPK pathway activity that was attenuated by *ATOX1* reduction. This shows that *ATOX1* is an important player that may have potential oncogenic properties.

One significant signaling cascade that controls several cellular functions, such as cell division, growth, proliferation, and responsiveness to external stimuli, is the MAPK pathway [40]. This pathway consists of a number of protein kinases that influence gene expression and cell behavior by transmitting signals from cell surface receptors to the nucleus. The MAPK pathway consists of three major kinases, namely, JNK, ERK, and p38 MAP kinase [41]. A sequence of phosphorylation events activates these kinases in response to a variety of stimuli, including growth factors, stress, cytokines, and hormones [42]. When active, the MAPK pathway performs a variety of functions, whereas the ERK pathway is largely engaged in cell proliferation, survival, and differentiation [43]. The JNK pathway is frequently activated by stress signals and plays a role in apoptosis, inflammation, and cellular damage responses [44]. The p38 pathway, which responds to numerous stimuli, regulates inflammation, apoptosis, and cell cycle arrest [45]. Abnormal regulation of the MAPK pathway is also linked with tumor. Research has demonstrated that via controlling the ERK/MAPK signaling pathway and specifically targeting MEK1, miR-101 regulates cell proliferation and apoptosis in DLBCL [46]. Another study found that *ATOX1* is necessary for MAPK pathway activation in melanoma, which indirectly supports MEK1/2 copper binding [47]. Through the WB method, we discovered that P-ERK1/2 protein expression within the MAPK pathway was significantly reduced in DLBCL cells when *ATOX1* was knocked down, which could be reversed by CuCl_2_. This highlights the intertwined roles of *ATOX1* and copper in regulating MAPK pathway activation. DCAC50 is a small molecule inhibitor; study has confirmed that it is related to the other partner protein ATOXA [48]. Our observations revealed that DCAC50 administration ensures a dose-dependent attenuation in DLBCL cell proliferation, with pronounced inhibition discernible at higher dosages, especially at 15 and 20 µM. The antiproliferative prowess of this compound seemingly hinges on its capacity to disrupt the MAPK pathway. Intriguingly, this inhibition is augmented when combined with an *ATOX1* knockdown, hinting at a synergistic relationship. The interplay between DCAC50 and the MAPK pathway, particularly in the context of *ATOX1*, not only broadens our understanding of DLBCL pathophysiology but also furnishes potential therapeutic avenues.

Despite the valuable insights gained from our study, it is important to acknowledge several limitations that may affect the interpretation and generalization of our findings. First, our conclusions were largely based on database and cell line data, which provided preliminary exploratory information. However, the lack of direct tissue-level validation is a major limitation. In future studies, we plan to validate the expression of *ATOX1* in different types of tissue samples by immunohistochemistry (IHC) experiments to provide stronger evidence to support our hypothesis and conclusions. Second, we have not yet validated the therapeutic effect of *ATOX1* in DLBCL in in vivo experiments. Although our study revealed the potential role of *ATOX1* in regulating the MAPK pathway through copper transport in DLBCL, these conclusions were only based on preliminary experimental data. To further delve into the study and validate our hypothesis, we plan to conduct detailed in vivo experiments in animal models of DLBCL to assess the specific role of *ATOX1* and its potential therapeutic effects. In conclusion, these limitations emphasized the need for further studies to validate our preliminary findings and to provide a stronger foundation for the future development of new therapeutic strategies. Furthermore, our study was a preliminary exploration of the unique role and innovative potential of the *ATOX1* gene in DLBCL. Future studies will focus on integrating other related cup mutated genes and using multivariate analysis methods to establish more comprehensive and accurate prognostic prediction models for DLBCL. This will help to provide clinicians with more effective support for individualized therapeutic decision-making, thereby maximizing patient clinical management and disease prognosis.

## Conclusion

To investigate the role of cuproptosis-related genes in DLBCL, we conducted molecular biology analysis. Our findings indicate that six genes are involved in the progression of DLBCL, with *ATOX1* being the most promising for disease diagnosis. In vitro cell experiments showed that *ATOX1* knockdown led to cell cycle arrest in the G2 phase and restricted the proliferation of DLBCL cells. Furthermore, this *ATOX1* knockdown resulted in decreased levels of P-ERK1/2 protein in the MAPK pathway, which were restored by CuC_2_ treatment. DCAC50 treatment showed dose-dependent antiproliferative effects, which were synergistically enhanced by *ATOX1* knockdown. These results suggest that the copper chaperone *ATOX1* may regulate copper transport to promote MAPK signaling and DLBCL growth, offering a new perspective on DLBCL prognosis.

## Supplemental data

**Figure S1.**
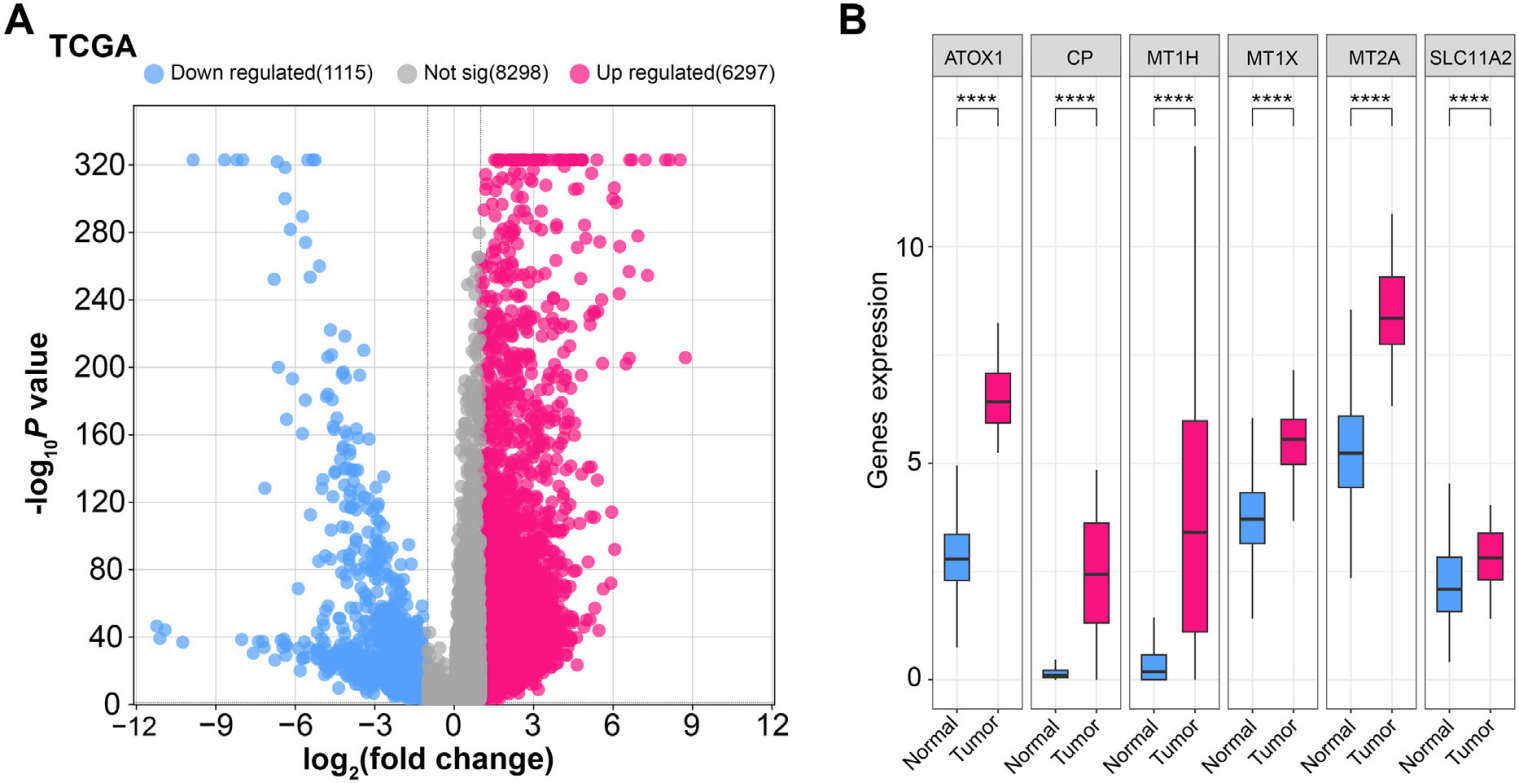
(A) Volcano plot showing differential gene expression in TCGA data: the plot illustrates the log_2_ fold change of gene expression against the −log_10_ P value for each gene. Blue dots represent 1115 downregulated genes, red dots represent 6297 upregulated genes, and gray dots represent 8298 genes with no significant change in expression. The *x*-axis indicates the log_2_ fold change, while the *y*-axis shows the −log_10_ *P* value. Genes with significant changes in expression are plotted on either side, with downregulated genes on the left and upregulated genes on the right. (B) Box plots of gene expression levels in normal and tumor samples: the plots compare the expression levels of ATOX1, CP, MT1H, MT1X, MT2A, and SLC11A2 genes between normal and tumor tissues. Each box plot displays the median gene expression (line within the box), interquartile range (box), and range (whiskers). Pink boxes represent tumor samples, and blue boxes represent normal samples. Asterisks above the boxes indicate significant differences in expression levels between normal and tumor tissues (**** *P* < 0.0001).

**Table S1:** Sequences of primer

**Gene name**	**Sequences of primer**	**Annealing temperature**	**Product length**
*ATOX1*	Forward: 5′-TCTGAGCACAGCATGGACACTC-3′	53°C	22bp
	Reverse: 5′-TCTGGAAGCCAGCGGGAGGAT-3′		
*GAPDH*	Forward: 5′-CAGTCAGCCGCATCTTCTTTTGCGTCG-3′	55°C	27bp
	Reverse: 5′-CAGAGTTAAAAGCAGCCCTGGTGACCAGG-3′		

Sequences of primers used for PCR amplification of the *ATOX1* and *GAPDH* genes. For each gene, the forward and reverse primer sequences are provided. The table also includes the annealing temperatures used during PCR and the length of the PCR product in base pairs (bp). The annealing temperatures for *ATOX1* and *GAPDH* primers are 53°C and 55°C, respectively, with product lengths of 22 bp for *ATOX1* and 27 bp for *GAPDH*.

## Data Availability

All data analyzed during this study are obtained from published article or are available from the corresponding author on reasonable request.
